# School-based health centers and inspiring the next generation of biomedical leaders

**DOI:** 10.1172/JCI196867

**Published:** 2025-07-15

**Authors:** Connie Cai

**Affiliations:** Johns Hopkins University School of Medicine, Baltimore, Maryland, USA.

The chairs in my office were far too small. My colleagues and I had to place two side-by-side, just to sit comfortably. But in the chairs’ defense, they weren’t designed for us –– they were made for five-year-olds.

For the day, my office was a kindergarten classroom. I was conducting vision screenings for students at KIPP Baltimore, a local charter school serving approximately 1,600 students from pre-K through eighth grade. These screenings were coordinated by the school-based health center (SBHC) located within the school. This SBHC was a fully operational clinic, with even an on-site lab. But beyond offering routine check-ups, this SBHC was a lifeline for the students and their community, offering school-wide vision screenings and well-child visits, administering vaccines, providing medications like inhalers and insulin, and educating both students, teachers, and families on topics ranging from asthma management to healthy cooking to mental health support.

During medical school, I co-founded the School Health Initiative, an organization that introduced medical students to the ways that health and education intersected. The connection between health and education is undeniable; healthier students perform better academically, and in turn, educational achievement has been shown to be correlated with better health (1). By providing accessible health care and health education, SBHCs have been shown to improve both health and educational outcomes (1). However, beyond improving outcomes, I believe that SBHCs have the potential to shape our future biomedical workforce for the better.

Increasingly, SBHCs are being recognized for their role in youth leadership and advocacy (2). As they have expanded, SBHCs are no longer just treating their students; they are also engaging students in their own health and in the health of their communities. At KIPP Baltimore, for example, our students participate in a Youth Advisory Council where they identify pressing health issues and help design programming to address them. This early exposure to health advocacy has a profound effect, planting the seeds for future careers in medicine and biomedical research. Studies show that early engagement in these health-related activities increases the likelihood of students pursuing biomedical careers, particularly among underrepresented minorities (3).

I have seen this firsthand in my work at MERIT Health Leadership Academy, a Baltimore-based nonprofit whose mission is to ensure that the biomedical workforce of tomorrow reflects the communities that it will serve. At MERIT, I was in charge of designing a career exploration curriculum for talented high school students. There, I met the next generation of biomedical engineers, physician scientists, cardiothoracic surgeons, dermatologists/skincare brand founders, and biotech venture capitalists. These were my students’ real career goals –– they weren’t just vague aspirations –– and they were committed to achieving them. Many of my students were in fact KIPP Baltimore alumni. They had once been patients at the SBHC, members of the Youth Advisory Council, and now they were training to become leaders in not just their school or community, but leaders in medicine.

This is how we build an inclusive and sustainable biomedical workforce. Not by starting at the graduate or even undergraduate level, but by investing in students early, and by giving them exposure to health care in a way that is meaningful, personal, and empowering.

As we conducted vision screenings in the kindergarten classroom, one of our patients, an adorable five-year-old boy, could not stay still. He bounced off the walls, the chairs, the bookshelves, you name it. When we were finally able to sit him down and test his vision, we suddenly realized why. He could barely see. His vision at five years old was worse than mine, and I had worn glasses since I was eight.

We handed him a pair of trial glasses, and for the first time that day, he stood still.

“You put medicine in these!” he shouted, taking the world in for the first time. A big grin spread across his face, as he reached out for a hug. “I want to be a doctor too!” he proclaimed.

I hope that in a few decades, when I’ll need the strongest reading glasses out there, he’ll be my ophthalmologist. Maybe he’ll even offer me the latest version of LASIK, or whatever corrective eye surgery he invents by then.

SBHCs are an untapped resource for building the future biomedical workforce. Research has shown that beyond serving underresourced communities and improving health outcomes, they also expose youth to careers in medicine and research (4). By expanding SBHCs, strengthening their youth engagement programs, and integrating mentorship pipelines like MERIT, we can foster a generation of diverse biomedical leaders who are not only equipped to succeed, but who also reflect the communities they serve.

## Version 1 07/15/2025

Electronic publication
